# Altered cerebral perfusion in Parkinson's disease patients with anxiety: an arterial spin labeling MRI study

**DOI:** 10.3389/fneur.2025.1583451

**Published:** 2025-05-15

**Authors:** Lu Li, Shiyuan Song, Yingying Hu, Yuan Luo, Lu Wang, Peiyao Zhang

**Affiliations:** ^1^Department of Radiology, China-Japan Friendship Hospital, Beijing, China; ^2^Department of Neurology, China-Japan Friendship Hospital, Beijing, China

**Keywords:** Parkinson's disease, anxiety, arterial spin labeling, magnetic resonance imaging, cerebral blood flow

## Abstract

**Purpose:**

In this study, we used arterial spin labeling (ASL) to explore altered cerebral blood flow perfusion in Parkinson's disease (PD) patients with anxiety and assessed the relationship between anxiety and perfusion in various brain regions to determine the pathophysiologic basis for the occurrence of anxiety in patients with PD.

**Materials and methods:**

Seventy-three patients with PD who were treated at China-Japan Friendship Hospital from September 2023 to November 2024 were enrolled: 36 PD patients with anxiety (PD-A) and 37 PD patients without anxiety (PD-NA); in addition, 37 healthy volunteers were recruited as healthy controls (HCs). All the subjects underwent three-dimensional T1-weighted imaging (3D-T1WI) and pseudo-continuous arterial spin labeling (pCASL) sequential scans via 3.0-T MRI, and cerebral blood flow (CBF) values were obtained from the whole brain. Independent samples *t* tests and non-parametric Mann–Whitney U tests were applied to test the differences in the CBF values of each brain region between the PD and HC groups, and between the PD-A and PD-NA groups. The relationships between CBF values and anxiety scores in the PD group were also investigated.

**Results:**

CBF values in the bilateral frontal lobes, parietal lobes, temporal lobes, occipital lobes, substantia nigra, striatum, caudate nuclei, left pallidum, and bilateral cerebellum were lower in the PD group than in the HC group (*P* < 0.05). Compared with those in the PD-NA group, the CBF values of the bilateral frontal lobes, temporal lobes, left putamen and left pallidum were lower in the PD-A group (*P* < 0.05). CBF values in the left frontal lobe (r = −0.265, *P* = 0.024), right frontal lobe (r = −0.283, *P* = 0.015), left temporal lobe (r = −0.287, *P* = 0.014), and right temporal lobe (r = −0.275, *P* = 0.019) were negatively correlated with Hamilton Anxiety Scale (HAMA) scores in PD patients.

**Conclusion:**

The development of PD-A may be associated with dysfunctional brain perfusion in multiple brain regions, notably the bilateral frontal lobes, temporal lobes, left putamen, and left pallidum. Abnormal CBF in these brain regions may serve as a neuroimaging marker for early PD-A diagnosis. Using ASL to identify perfusion changes in core regions may advance our understanding of the pathophysiological mechanisms underlying PD-A.

## 1 Introduction

Parkinson's disease (PD) is the second most common neurodegenerative disorder (1) and is characterized by bradykinesia, rigidity, resting tremor, postural instability, gait impairment, and numerous non-motor and behavioral symptoms (2). Cognitive impairment and psychiatric disorders are common non-motor symptoms that may manifest in early stages of the disease, sometimes even before the appearance of classic motor symptoms (3, 4). Furthermore, the onset and progression of non-motor symptoms have a greater impact on PD patients than motor symptoms do (5). Anxiety is one of the most common non-motor symptoms in PD patients, and the prevalence of PD with anxiety (Parkinson's disease patients with anxiety [PD-A]) is approximately 31% (6), which affects patient's quality of life. The neuropathological hallmarks of PD are neuronal loss in the substantia nigra, which causes striatal dopaminergic deficiency, and α-synuclein accumulation in intraneuronal inclusions (1). The underlying mechanisms of PD-A remain largely unknown, and exploration of their neurobiological alterations can assist in the further diagnosis and treatment of PD-A patients.

Arterial spin labeling (ASL) is a noninvasive magnetic resonance imaging (MRI) technique that uses magnetically labeled blood water as an endogenous tracer for perfusion measurements (7). The absence of dopaminergic neurons alters the microvascular environment and affects cerebral blood perfusion (8). Recent studies have demonstrated that PD patients exhibit cortical hypoperfusion in brain regions that are associated with motor and non-motor symptoms (9–11). Reduced CBF in PD patients is an important measure for assessing motor symptoms, non-motor symptoms, and dopamine treatment (12, 13). Prior investigations of cerebral perfusion in PD-A patients have focused predominantly on single-photon emission computed tomography (SPECT) (14). SPECT-based imaging necessitates the use of radiotracers, which may pose potential risks and limitations related to invasiveness and radiation exposure. In contrast, ASL is a non-invasive, radiation-free technique that offers enhanced safety, faster acquisition times, and superior reproducibility. These attributes render ASL a promising modality by which to elucidate regional perfusion alterations in PD-A patients.

In this study, we used the ASL technique to explore cerebral perfusion in PD-A patients and to assess the relationship between anxiety scores and CBF values to determine the pathophysiologic basis for the development of anxiety in PD patients.

## 2 Materials and methods

### 2.1 Participants

This prospective study was approved by the Ethics Committee of the China-Japan Friendship Hospital. All participants were informed of the study details and methods and provided written informed consent. A total of 81 patients with PD who were treated at China-Japan Friendship Hospital between September 2023 and November 2024 were prospectively enrolled. The inclusion criteria were as follows: (1) diagnosis and evaluation by two experienced neurologists according to the Movement Disorder Society (MDS) clinical diagnostic criteria for Parkinson's disease; (2) modified Hoehn & Yahr (H&Y) stage of 1–2.5; and (3) right-handedness. The exclusion criteria were as follows: (1) history of head trauma, stroke, central nervous system infection, or tumor (*n* = 3); (2) contraindications to MRI examination, such as claustrophobia or the presence of metallic implants (*n* = 2); (3) significant motion artifacts in the scan images (*n* = 3); and (4) severe psychiatric or cognitive disorders. Ultimately, 73 PD patients met the inclusion criteria and were enrolled in the study.

Thirty-seven healthy volunteers were recruited from the community to serve as the healthy controls (HCs). The inclusion criteria for the HCs were as follows: (1) no history of traumatic brain injury or stroke and no psychiatric or cognitive disorders; (2) Hamilton Anxiety Scale (HAMA) score <7; (3) right-handedness; and (4) no contraindications to MRI examination.

### 2.2 Clinical information collection and neuropsychological scale assessment

PD patients were evaluated by senior neurologists with specialized training on the basis of the diagnostic criteria for anxiety attacks outlined in the Diagnostic and Statistical Manual of Mental Disorders, 4th edition (DSM-IV). PD patients were categorized into two groups according to HAMA score: the PD-A group and the PD-NA group. Specifically, a HAMA score ≥ 7 was used to identify PD patients with anxiety (PD-A, *n* = 36), whereas those with a HAMA score <7 were classified as PD patients without anxiety (PD-NA, *n* = 37). The severity of PD was assessed using the H&Y staging system, and cognitive function was evaluated using the Mini-Mental State Examination (MMSE). The motor function of PD patients was assessed with the MDS modified version of the Unified Parkinson's Disease Rating Scale motor examination (UPDRS-III).

### 2.3 MRI data acquisition and processing

All participants completed MRI examinations on a 3.0-T MR scanner (GE Healthcare, Discovery MR 750, United States) with an 8-channel head coil. All PD patients discontinued any dopamine replacement therapy for at least 48 h prior to evaluation. The participants were instructed to keep their eyes closed, their bodies relaxed, and their heads still during the scanning process. The scanning sequences included three-dimensional T1-weighted imaging (3D-T1WI) and pseudo-continuous arterial spin labeling (pCASL). Three-dimensional T1-weighted imaging (3D-T1WI) were acquired with the following parameters: repetition time (TR), 6.7 ms; echo time (TE), 2.5 ms; flip angle, 12°; slice thickness, 1 mm; number of slices, 192; and field of view (FOV), 256 mm × 256 mm. The perfusion-weighted images were acquired at pCASL with a post label delay (PLD) of 2,025 ms. The scan parameters were as follows: TR, 5,029 ms; TE, 14.6 ms; bandwidth, 62.5 kHz; slice thickness, 4 mm; number of slices, 36; and FOV, 240 mm × 240 mm.

The ASL data were post processed via CereFlow software (Translational MRI, LLC, Los Angeles, CA, United States) using the following steps: (1) The raw ASL data and CBF data were converted into parametric cerebral perfusion maps to obtain the mCBF (mean cerebral blood flow). (2) The brain perfusion parameter maps were normalized to the Montreal Neurological Institute (MNI) standard brain templates, resampled after normalization, and spatially smoothed. (3) Brain mapping of the arterial blood supply region and automatic anatomical labeling brain mapping for overlay were conducted. (4) The mean value of perfusion within each partitioned volume was obtained. The perfusion regions of interest (ROIs) included the frontal lobe, parietal lobe, temporal lobe, occipital lobe, substantia nigra, striatum, caudate, putamen, pallidum, and cerebellum.

### 2.4 Statistical analysis

Statistical analysis was conducted using SPSS 26.0 (IBM, Armonk, New York, USA). Categorical data, such as gender, are expressed as the number of cases, and comparisons among the three groups were performed using the χ^2^ test. For continuous data (age, scale scores, and CBF values), data that conformed to a normal distribution were described as the mean ± standard deviation (x̄ ± s), whereas those not conforming to a normal distribution were described as the median and interquartile range (M, P25–P75).

Comparisons between two groups for normally distributed variables were performed using the independent samples *t* test, whereas comparisons among multiple groups were conducted using one-way analysis of variance (ANOVA). For non-normally distributed variables, comparisons between two groups were performed using the Mann–Whitney U test, and comparisons among multiple groups were conducted using the Kruskal–Wallis H test.

Spearman correlation analysis was employed to assess the relationships between HAMA scores and CBF values in different brain regions among PD patients. All hypothesis tests were two-sided, with *P* < 0.05 indicating statistical significance.

## 3 Results

### 3.1 Analysis of clinical data

There were no statistically significant differences among the three groups (HC, PD-NA, and PD-A) in terms of age, gender, MMSE score, or Hamilton Depression Scale (HAMD) score (*P* > 0.05). Additionally, no significant differences were observed between the PD-NA and PD-A groups regarding disease duration, UPDRS-III score, or H&Y stage (*P* > 0.05). In terms of the HAMA score, no significant difference was found between the HC and PD-NA groups (*P* > 0.05). However, significant differences were observed between the HC and PD-A groups, as well as between the PD-NA and PD-A groups (*P* < 0.05). Specifically, the HAMA scores of the PD-A group were higher than those of the HC and PD-NA groups. Detailed results are presented in Table 1.

**Table 1:** Demographic and clinical characteristics of the samples.

**Groups**	**HC (*n =* 37)**	**PD-NA (*n =* 37)**	**PD-A (*n =* 36)**	**χ^2^/*t*/*F*/*Z***	***P***
Gender (M/F)	12/25	17/20	14/22	1.420^a^	0.492
Age (years)	63.24 ± 8.37	65.54 ± 10.55	63.42 ± 9.16	0.682^b^	0.508
MDS-UPDRS-III score	-	21.38 ± 13.31	28.83 ± 12.93	−2.427^c^	0.018
Disease course (months)	-	36.0 (24.0, 48.0)	36.0 (15.0, 60.0)	−0.067^d^	0.947
Hoehn & Yahr stage	-	2.0 (1.0, 2.0)	2.0 (1.5, 2.4)	−1.414^d^	0.157
HAMA score	3.0 (2.0, 5.0)	3.0 (1.0, 5.0)	10.0 (8.3, 13.8)^*#^	72.621^e^	<0.001
MMSE score	29.0 (27.0, 30.0)	29.0 (27.0, 30.0)	28.0 (26.3, 29.0)	2.456^e^	0.293
HAMD score	3.0 (2.0, 4.0)	3.0 (2.0, 5.0)	3.0 (2.0, 4.0)	−0.992^e^	0.321

^*^Indicates a statistically significant difference compared with HCs (*P* < 0.05), ^#^indicates a statistically significant difference compared with PD-NA patients (*P* < 0.05). ^a^χ^2^ test; ^b^one-way ANOVA; ^c^two independent samples *t-*test; ^d^Mann–Whitney U test; ^e^Kruskal–Wallis H test. HC, healthy control; PD-NA, Parkinson's disease without anxiety; PD-A, Parkinson's disease with anxiety.

### 3.2 Comparison of CBF values

**Figure 1:**
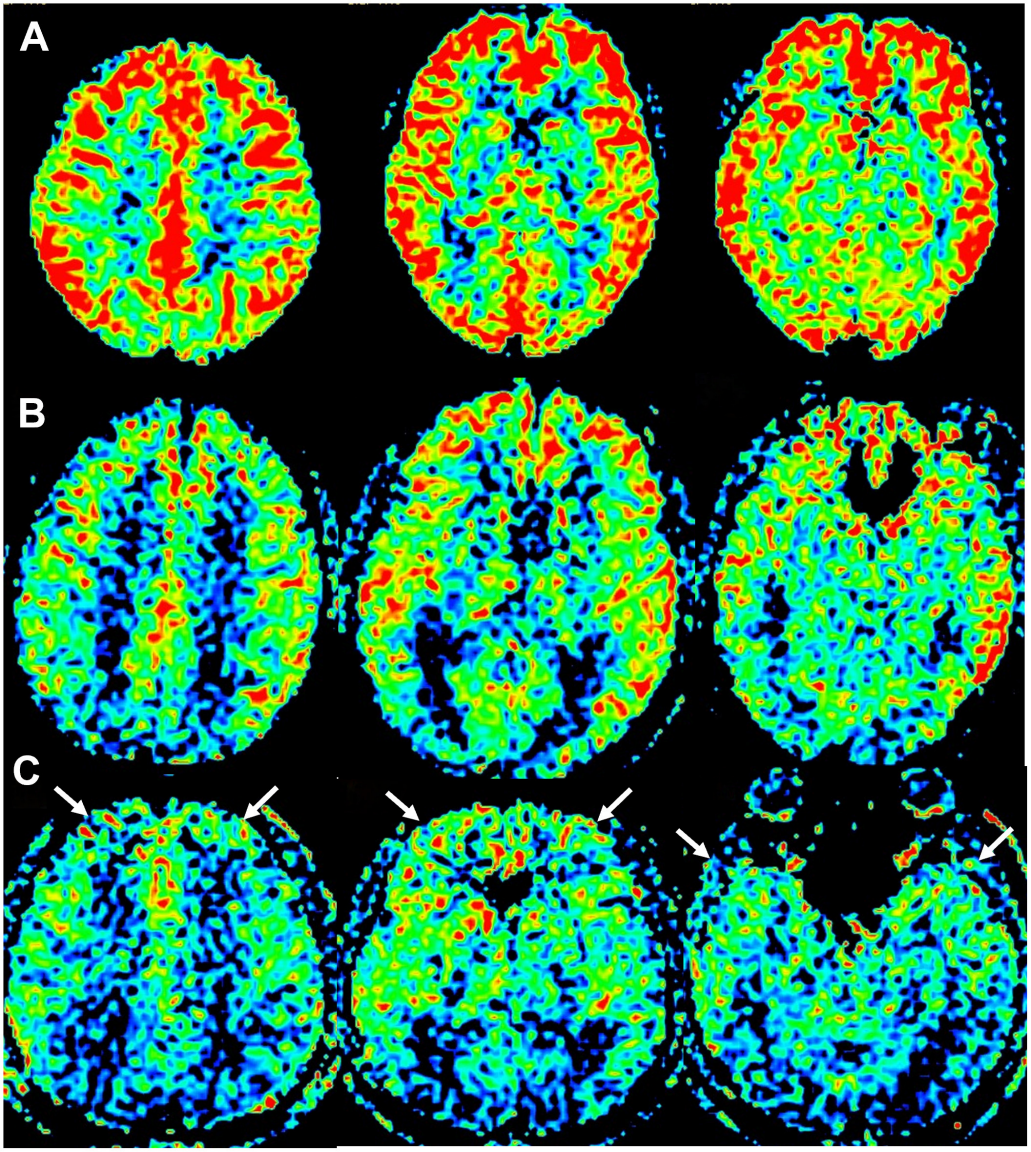
Fused images of cerebral blood flow (CBF). **(A)** Image of a healthy control (HC) participant (female, 55 years old). **(B)** Image of a participant in the Parkinson's disease without anxiety (PD-NA) group (male, 51 years old). **(C)** Image of a participant in the Parkinson's disease with anxiety (PD-A) group (female, 66 years old). Compared to the HC group **(A)**, both PD-NA **(B)**, and PD-A **(C)** demonstrate widespread hypoperfusion in multiple brain regions. The PD-A group **(C)** exhibits further reduced perfusion in bilateral frontal and temporal lobes compared to PD-NA **(B)** (white arrows).

Compared with the HCs, the PD patients presented reduced CBF values in multiple brain regions, including the bilateral frontal lobes, parietal lobes, temporal lobes, occipital lobes, substantia nigra, striatum, caudate nuclei, left pallidum, and bilateral cerebellum (Figure 1, Table 2).

**Table 2:** Brain regions with perfusion differences between the HC group and the PD group.

**Brain regions**	**HC (*n =* 37)**	**PD (*n =* 73)**	***t*/*Z***	***P***
Frontal L	53.87 (49.34, 59.54)	48.49 (41.69, 51.95)	25.523	<0.001^*^
Frontal R	48.25 (45.79, 54.87)	44.41 (40.03, 49.29)	18.908	<0.001^*^
Parietal L	51.83 (47.22, 56.13)	43.33 (37.15, 49.42)	29.028	<0.001^*^
Parietal R	47.19 (44.22, 50.65)	39.15 (32.42, 44.15)	30.781	<0.001^*^
Temporal L	54.26 (50.37, 56.69)	46.33 (41.53, 50.84)	28.840	<0.001^*^
Temporal R	52.13 (48.95, 55.74)	44.56 (39.00, 50.12)	31.592	<0.001^*^
Occipital L	55.47 (51.00, 62.17)	46.05 (37.98, 51.38)	30.924	<0.001^*^
Occipital R	54.36 (50.30, 59.22)	46.04 (37.77, 51.33)	30.015	<0.001^*^
Substantia Nigra L	43.76 ± 6.57	40.29 ± 7.47	2.393	0.018^*^
Substantia Nigra R	44.50 ± 6.94	40.93 ± 6.94	2.552	0.012^*^
Striatum L	37.98 (35.29, 39.79)	34.93 (33.01, 38.26)	10.757	0.005^*^
Striatum R	39.29 (36.62, 43.51)	38.27 (34.68, 41.57)	6.337	0.042^*^
Caudate L	33.83 (31.91, 37.99)	30.37 (27.49, 34.92)	11.527	0.003^*^
Caudate R	38.89 (35.60, 42.01)	35.99 (32.18, 38.75)	10.289	0.006^*^
Putamen L	41.44 ± 5.86	40.01 ± 5.18	1.309	0.193
Putamen R	41.84 ± 6.30	40.05 ± 5.19	1.588	0.115
Pallidum L	35.73 (33.39, 38.49)	34.36 (32.20, 38.52)	6.303	0.043^*^
Pallidum R	37.95 (35.79, 44.29)	38.08 (35.49, 42.75)	1.771	0.412
Cerebellum L	54.59 ± 10.13	46.33 ± 8.76	4.428	<0.001^*^
Cerebellum R	55.08 ± 10.40	46.71 ± 8.83	4.419	<0.001^*^

^*^Statistically significant difference (*P* < 0.05), PD, Parkinson's disease patients.

Further comparisons revealed that PD-A patients presented significantly lower perfusion in the bilateral frontal lobes, temporal lobes, left putamen, and left pallidum than PD-NA patients (Figure 1, Table 3).

**Table 3:** Brain regions with perfusion differences between the PD-NA group and the PD-A group.

**Brain regions**	**PD-NA (*n =* 37)**	**PD-A (*n =* 36)**	***t/Z***	***P***
Frontal L	49.39 ± 7.03	45.62 ± 7.06	2.284	0.025^*^
Frontal R	46.36 ± 6.98	42.3 ± 6.56	2.564	0.012^*^
Parietal L	44.62 ± 8.51	41.56 ± 7.95	1.586	0.117
Parietal R	40.14 ± 8.12	36.9 ± 7.93	1.723	0.089
Temporal L	48.70 ± 7.70	44.44 ± 7.03	2.468	0.016^*^
Temporal R	46.82 ± 7.76	42.42 ± 7.13	2.519	0.014^*^
Occipital L	46.12 ± 9.26	42.79 ± 10.25	1.460	0.149
Occipital R	47.91 (38.61, 53.71)	43.27 (36.01, 49.97)	−1.589	0.112
Substantia Nigra L	40.20 ± 7.53	40.39 ± 7.52	−0.108	0.914
Substantia Nigra R	41.98 ± 6.55	39.85 ± 7.26	1.315	0.193
Striatum L	36.46 ± 5.57	34.56 ± 4.11	1.653	0.103
Striatum R	38.63 ± 4.92	37.43 ± 4.78	1.055	0.295
Caudate L	31.44 ± 6.90	30.91 ± 5.00	0.376	0.708
Caudate R	35.56 ± 5.67	35.26 ± 4.98	0.241	0.810
Putamen L	41.32 ± 5.26	38.67 ± 4.80	2.254	0.027^*^
Putamen R	40.84 ± 5.08	39.23 ± 5.25	1.336	0.186
Pallidum L	37.00 ± 4.93	34.27 ± 4.36	2.507	0.014^*^
Pallidum R	40.05 ± 5.80	38.3 ± 5.59	1.309	0.195
Cerebellum L	47.65 ± 9.10	44.97 ± 8.30	1.314	0.193
Cerebellum R	48.71 ± 8.56	44.66 ± 8.74	1.998	0.052

^*^Statistically significant difference (*P* < 0.05).

### 3.3 Correlation between the CBF value and HAMA score

**Figure 2:**
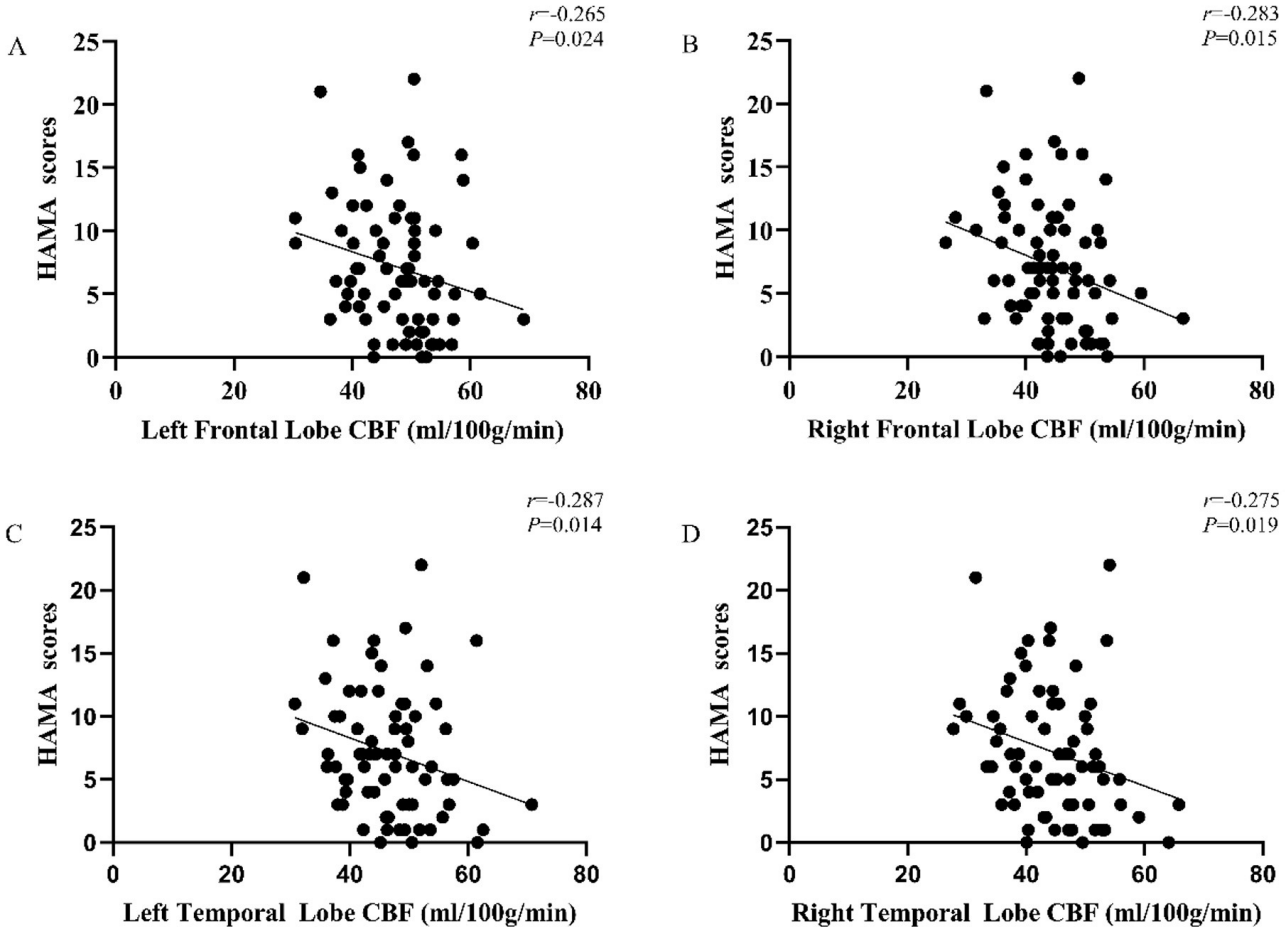
Correlation between CBF value and Hamilton Anxiety Scale (HAMA) score in PD patients. **(A)** The CBF values of left frontal lobe were negatively correlated with the HAMA scores (r = −0.265, *P* = 0.024); **(B)** the CBF values of right frontal lobe were negatively correlated with the HAMA scores (r = −0.283, *P* = 0.015); **(C)** the CBF values of left temporal lobe were negatively correlated with the HAMA scores (r = −0.287, *P* = 0.014); **(D)** the CBF values of right temporal lobe were negatively correlated with the HAMA scores (r = −0.275, *P* = 0.019).

The HAMA score and CBF value of each brain region in the PD group were analyzed for correlation, and the results indicated that the HAMA scores were negatively correlated with the CBF values in the left frontal lobe (r = −0.265, *P* = 0.024), right frontal lobe (r = −0.283, *P* = 0.015), left temporal lobe (r = −0.287, *P* = 0.014), and right temporal lobe (r = −0.275, *P* = 0.019), as shown in Figure 2 and Table 4.

**Table 4:** Correlation analysis between the CBF values and HAMA scores in different brain regions.

**Brain regions**	**HAMA scores**	
	**r-value**	***P*** **value**
Frontal L	−0.265	0.024^*^
Frontal R	−0.283	0.015^*^
Parietal L	−0.228	0.052
Parietal R	−0.222	0.059
Temporal L	−0.287	0.014^*^
Temporal R	−0.275	0.019^*^
Occipital L	−0.163	0.168
Occipital R	−0.197	0.095
Substantia Nigra L	−0.081	0.494
Substantia Nigra R	−0.173	0.144
Striatum L	−0.026	0.829
Striatum R	−0.067	0.575
Caudate L	0.045	0.708
Caudate R	0.005	0.969
Putamen L	−0.117	0.324
Putamen R	−0.102	0.389
Pallidum L	−0.161	0.174
Pallidum R	−0.094	0.427
Cerebellum L	−0.120	0.311
Cerebellum R	−0.208	0.077

^*^Statistically significant difference (*P* < 0.05).

## 4 Discussion

ASL is a brain imaging technique that uses labeled blood water from cerebral supply arteries as an endogenous tracer to obtain information about relevant cerebral perfusion and offers a non-invasive and highly reproducible method for assessing cerebral hemodynamics (15–17). In this study, we assessed alterations in the perfusion of brain regions between PD patients and healthy controls via the ASL technique. Our results revealed multiple brain regions with reduced perfusion in PD patients. Further analysis was conducted to compare perfusion patterns between PD-A and PD-NA patients. We found significant reductions in perfusion within the bilateral frontal lobes, temporal lobes, left putamen, and left pallidum in the PD-A group compared with the PD-NA group. Additionally, the CBF values in the bilateral frontal and temporal lobes of PD patients were negatively correlated with their HAMA scores. Through this study, we explored the neurological alterations associated with anxiety in PD patients by examining regional perfusion differences across groups, thereby providing imaging-based evidence to elucidate the underlying mechanisms involved.

In the present study, PD patients exhibited widespread cerebral hypoperfusion compared with HCs in the bilateral frontal lobes, parietal lobes, temporal lobes, occipital lobes, substantia nigra, striatum, caudate nuclei, left pallidum, and bilateral cerebellum, findings that are largely consistent with prior research (18–21). A study by Lin et al. (18) demonstrated extensive perfusion deficits in the bilateral frontal, parietal, and temporal lobes of PD patients. Cheng et al. (19) reported reduced CBF values in the left frontal lobe, right cerebellum and left caudate nucleus in PD patients, which are partially consistent with our findings. Melzer et al. (20) characterized metabolic and perfusion abnormalities in patients with PD and reported significant reductions in cerebral perfusion within the parieto-occipital cortex, cuneus, precuneus, and middle frontal gyrus, whereas smaller decreases were noted in additional cortical areas (parietal, frontal, and temporal regions) and subcortical structures (thalamus and caudate nucleus). Syrimi et al. (21) investigated the perfusion basis of cognitive changes in PD patients without dementia and reported decreased CBF in the posterior parietal lobe, precuneus, and posterior cingulate gyrus. Our findings partially align with these findings, as most brain regions in PD patients exhibit a hypoperfusion pattern, with only the right pallidum showing mildly increased perfusion. However, some previous studies are somewhat inconsistent with the present study (22, 23). Wang et al. (22) reported reduced CBF in the frontal, parietal, and occipital lobes of PD patients, whereas CBF was increased in the thalamus, and chiasmatic nucleus. Similarly, Liu et al. (23) reported lower CBF in the frontal, parietal, and temporal lobes of PD patients than in those of healthy volunteers, whereas the CBF was greater in the bilateral hippocampus, red nucleus, right substantia nigra, thalamus, and most cerebellar regions. The discrepancy between our results and those of prior ASL studies may be due to differences between the subject groups. Liu's research focused on advanced PD patients (H&Y ≥ 3), whereas our subjects were early-stage PD patients. Advanced PD may exhibit compensatory phenomena in certain brain regions characterized by hypoperfusion in cortical regions and hyperperfusion in subcortical and cerebellar regions (23). The cerebellar cortex may play an important role in both motor and non-motor performance. For example, lobules I–V of the anterior lobe, adjacent parts of lobule VI and lobule VIII of the posterior lobe support sensorimotor function. Lobules VI and VII support cognitive functions (24, 25). The primary pathological hallmark of PD is the degeneration of nigrostriatal dopaminergic neurons, which disrupts the normal functioning of basal ganglia circuits. Dopaminergic neuronal loss modifies the microvascular environment and may affect cerebral blood perfusion. Neurodegeneration and reduced metabolism may subsequently disturb CBF (26). In PD patients, impaired motor and cognitive functions are influenced by the cerebello-thalamo-cortical (CTC) circuit and the striatal-thalamo-cortical (STC) circuit. Dopaminergic deficits may lead to the suppression of the function of these circuits, which can result in a decrease in metabolic demand and a reduction in CBF in relevant brain regions. Additionally, dopaminergic medications are commonly used in PD patients and their effects on cerebral blood flow warrant further investigation. Hershey et al. (27) concluded that levodopa had a minimal effect on cerebral blood flow perfusion in PD patients undergoing initial levodopa treatment. In contrast, Xiong et al. (28) recently reported that levodopa could increase CBF values in corresponding brain regions by dilating intracranial arteries. In order to minimize the influence of medication on the results of this study, the PD subjects included in our study discontinued dopaminergic medications for 48 h prior to evaluation. Differences in the results of relevant clinical studies may also be related to variations in disease duration, disease staging, and drug administration between subjects.

In this study, we investigated the differences in perfusion between PD-A and PD-NA patients. Compared with the PD-NA group, the PD-A group presented reduced perfusion in the bilateral frontal lobes, temporal lobes, left putamen, and left pallidum. Additionally, correlation analyses revealed that perfusion values in the bilateral frontal and temporal lobes were negatively correlated with HAMA scores, which suggests that these regions may serve as key brain regions involved in the pathophysiology of anxiety-related mechanisms. The prefrontal cortex, a key brain region associated with emotional disorders, is crucial for functions such as decision-making, emotional regulation, and social cognition (29). A longitudinal study suggested that reduced prefrontal perfusion may underlie the progression of cognitive impairment during the early stages of PD (30). Zhang et al. (31) demonstrated that patients with PD-A exhibit significantly reduced levels of oxygenated hemoglobin in the left inferior frontal gyrus (IFG), which is negatively correlated with the severity of anxiety symptoms. The pathological mechanisms of anxiety in PD patients are closely associated with structural and functional abnormalities in the temporal lobe, particularly neurodegenerative changes and hemodynamic disturbances in the medial temporal lobe (e.g., hippocampus and amygdala) and lateral temporal regions (e.g., superior temporal gyrus and temporal pole). Structural magnetic resonance imaging (sMRI) studies have demonstrated that decreased gray matter volume (GMV) in the frontal and temporal lobes is associated with PD-A patients compared to PD patients without anxiety, indicating that these regions are involved in emotional regulation and cognitive control functions (32, 33). Deep brain stimulation (DBS) studies have demonstrated that the functional connectivity (FC) between specific brain regions (e.g., olfactory cortex- inferior frontal gyrus pars orbitalis connectivity and inferior temporal gyrus- posterior orbital gyrus connectivity) is significantly correlated with anxiety improvement rates in PD-A patients, which indicates that these regions play critical roles in both the generation and progression of anxiety symptoms (34, 35). Cortical structural and functional abnormalities in the frontal and temporal lobes may lead to changes in CBF in corresponding brain regions that are associated with cognitive and non-motor symptoms in PD patients. Vriend et al. (36) reported that the severity of anxiety symptoms in PD patients is associated with decreased availability of dopamine transporter proteins in the striatum. A positron emission tomography (PET) correlation study showed that changes in dopamine levels at different sites elicit distinct mood alterations. Specifically, reduced dopamine in the ventral striatum is correlated with the severity of apathy, whereas decreased dopamine level within the limbic system is often associated with the onset of anxiety (37). Anxiety symptoms in PD patients are negatively correlated with dopamine transporter density in the bilateral pallidum and the left putamen (38), which suggests that dysfunction in the pallidum and putamen may contribute to anxiety development through neurotransmitter imbalance or local blood flow changes. Anxiety may be associated with dysfunction in the limbic cortico-striato-thalamocortical circuits (6), which may exacerbate hypoperfusion in associated brain regions through neurotransmitter imbalance or changes in metabolic demands.

This study has several limitations. First, this study is limited by its cross-sectional, single-center design with a relatively small sample size. Therefore, validation of the current findings through a multicenter, large-sample longitudinal study is essential. Additionally, due to inherent limitations in the accuracy of scale assessments, only patients with early to mid-stage PD were included. Further investigations are warranted to determine the consistency of these findings in patients with advanced PD.

## 5 Conclusion

In this study, we applied ASL technology to investigate the differences in cerebral perfusion between PD patients and HCs, as well as between PD patients with and without anxiety. Our results demonstrated that PD patients exhibit significant alterations in cerebral perfusion, which are predominantly characterized by a hypoperfusion pattern. Specifically, the reduction in perfusion in the bilateral frontal and temporal lobes is correlated with the occurrence of anxiety in PD patients. These findings will help to provide a theoretical basis for the study of the pathophysiological mechanisms underlying anxiety in PD.

## Data Availability

The original contributions presented in the study are included in the article/supplementary material, further inquiries can be directed to the corresponding author.
